# Single-center experience of interventional therapy for congenital portal-systemic shunt in children

**DOI:** 10.3389/fped.2026.1795636

**Published:** 2026-07-01

**Authors:** Xiangfeng Guo, Qi Di, Gang Shen

**Affiliations:** Vascular Anomalies and Vascular Interventional Center, Capital Center for Children’s Health, Capital Medical University, Capital Institute of Pediatrics, Beijing, China

**Keywords:** children, congenital portosystemic shunt, interventional embolization, short-term efficacy, single-center

## Abstract

**Objective:**

To investigate the safety and short-term efficacy of interventional therapy for congenital portosystemic shunt (CPSS) in children, and to provide single-center small-sample empirical data for clinical practice.

**Methods:**

A retrospective analysis was conducted on the clinical data of 26 children with CPSS who underwent interventional therapy at Capital Center for Children's Health, Capital Medical University from January 2022 to August 2025. Based on anatomical subtypes, they were categorized into the intrahepatic portal-systemic shunt group (18 cases) and the Abernethy malformation group (8 cases). Techniques such as coil embolization and Amplatzer occluder embolization were employed. Postoperative follow-up was performed for 1–12 months to evaluate technical success rates, complication rates, and improvements in liver function and blood ammonia levels.

**Results:**

The success rate of interventional therapy in 26 pediatric patients was 73% (19/26). Among them, the technical success rate in the intrahepatic portal-systemic shunt group was 94.4% (17/18), while the Abernethy malformation technique achieved a success rate of 25% (2/8). The incidence of mild complications within 1 month postoperatively was 3.8% (1/26), including 1 case of puncture site hematoma, with no severe complications reported. Among the successfully treated patients, the normalization rate of postoperative liver function parameters [Alanine aminotransferase (ALT), Aspartate aminotransferase (AST)] was 63% (12/19), the improvement rate of bile acids was 73.7% (14/19), and the improvement rate of blood ammonia was 100% (19/19).

**Conclusion:**

The individualized interventional embolization strategy for children with different anatomical subtypes of CPSS is safe and effective, with definite short-term efficacy, and can be used as the first-line treatment for CPSS children.

Congenital portosystemic shunts (CPSS) are rare congenital disorders caused by developmental abnormalities of the portal venous system during the embryonic period. These malformations lead to the bypassing of hepatic blood flow into the systemic circulation, which may result in severe complications such as hepatic encephalopathy and hepatopulmonary syndrome if persisting long-term. CPSS are typically solitary malformations, though multiple shunts may also occur (1). CPSS are classified into intrahepatic portosystemic shunts (congenital intra-hepatic portosystemic shunt, CIPSS) and extrahepatic portosystemic shunts (congenital extra-hepatic portosystemic shunt, CEPSS). Although their clinical manifestations may be similar, the pathophysiology and treatment of these two types differ. Traditional surgical interventions were previously limited in clinical application due to their high trauma and postoperative risk of portal hypertension, whereas interventional therapy has become the preferred treatment owing to its minimally invasive advantages. From 2022 to 2025, our hospital treated 26 pediatric patients with CPSS, all of whom underwent personalized interventional therapy. This study retrospectively analyzed clinical data to summarize personalized interventional treatment strategies for CPSS patients and provide single-center therapeutic experience.

## 1 Materials and methods

### 1.1 General information

A retrospective study was conducted on 26 children with CPSS (Cerebral Perfusion Syndrome) admitted to our department from January 2022 to August 2025. Inclusion criteria: ① Age ≥1 and ≤18 years, with confirmed CPSS by contrast-enhanced Computed Tomography Angiography (CTA) or ultrasound; ② Eligibility for interventional therapy; ③ Informed consent signed by the guardian. Exclusion criteria: Patients with concurrent cirrhosis, portal vein thrombosis, severe cardiac insufficiency, or contrast agent allergy.

Among the 26 pediatric patients, 14 were male and 12 were female, with ages ranging from 1 to 16 years (mean: 5.88 ± 4.17 years). Anatomical subtypes: Abernethy type I in 6 cases, type II in 2 cases (1 case of superior mesenteric vein-left renal vein shunt and 1 case of main portal vein-left iliac vein shunt); intrahepatic portal-portal shunts in 17 cases (1 case of left hepatic branch-down inferior vena cava shunt, 7 cases of left hepatic branch-left hepatic vein shunt, 2 cases of main portal vein-down inferior vena cava shunt, and 8 cases of right hepatic branch-right hepatic vein shunt). Preoperative complications: abnormal liver function in 8 cases, pulmonary hypertension in 3 cases, hepatopulmonary syndrome in 1 case, and portal hypertension in 1 case (Table 1).

**Table 1:** General information.

General information	Number of patients
Gender	
Male	14
Female	12
Age	
1–5 years old	13
5–10 years old	8
10–15 years old	4
15–18 years old	1
Operation time	
≦100min	4
100–200min	16
≧200min	6
Shunt type	
Intrahepatic	18
Extrahepatic	8
Intrahepatic shunt types	
Left portal branch—inferior vena cava shunt	1
Left portal branch—left hepatic vein shunt	7
The main portal vein—inferior vena cava shunt	2
Right portal branch—right hepatic vein shunt	8
Extrahepatic shunt type	
Abernethy type I	6
Abernethy type II	2
Associated malformations	
Liver space-occupying lesion	4
Pulmonary arteriovenous fistula	1
Lateral branches of the body lung	1
Congenital heart disease	1
Preoperative complications	
Abnormal liver function	8
Pulmonary arterial hypertension	3
Hepatopulmonary syndrome	1
Portal hypertension	1

### 1.2 Methods

#### 1.2.1 Indications for surgery

1. Preoperative ultrasound and portal venous contrast-enhanced CTA confirming portal-portal shunting with clinical symptoms (hepatic encephalopathy, hepatopulmonary syndrome, portal hypertension, hepatic nodules);
2. Preoperative ultrasound and portal venous contrast-enhanced CTA confirming portal-portal shunting with the child aged ≥1 year;
3. Intraoperative indirect portal venography demonstrating intrahepatic portal venous imaging with portal pressure ≤26 cmH_2_O allows for shunt embolization.

#### 1.2.2 Preoperative assessment

1. Imaging: Portal CTA 3D reconstruction was performed to determine the diameter and course of the shunt tract, while ultrasonography of the portal system measured the shunt flow velocity and calculated the shunt index.
2. Hemodynamics: Measurement of portal venous pressure (PVP) before embolization, with an average value of (17.40 ± 4.21) cmH_2_O.

For cases scheduled for complete embolization, a temporary balloon occlusion test should be performed to ensure that the PVP elevation is ≤26 cmH_2_O.

3. Laboratory: Complete ALT, AST, blood ammonia, and coagulation function tests.

#### 1.2.3 Individualized interventional therapy strategies

**Figure 1:**
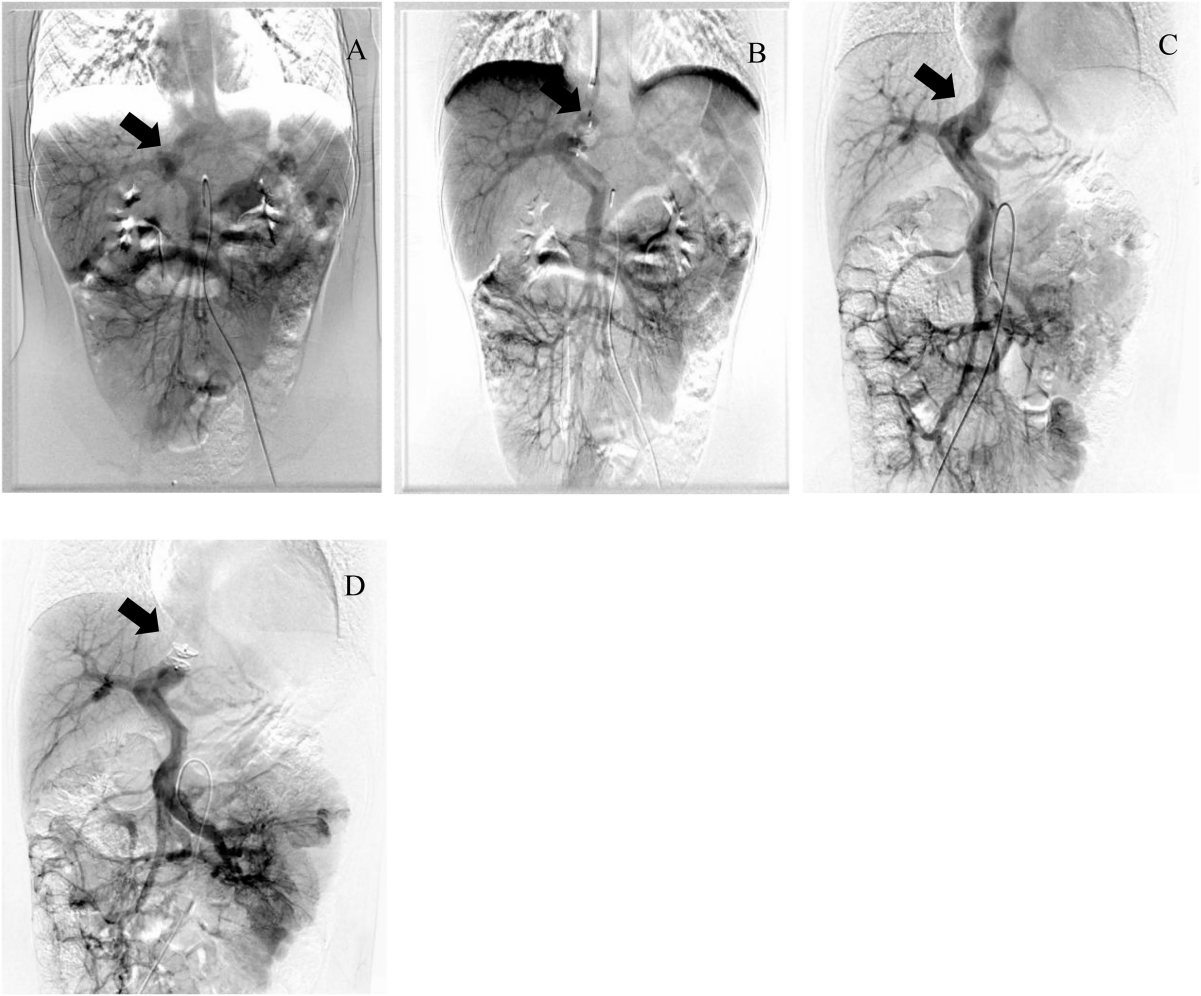
**(A,B)** A 5-year-old girl with intrahepatic portal-venous shunt detected during physical examination. Preoperative angiography revealed a shunt between the left portal branch and the left hepatic vein. Intraoperative vascular occlusion with a vascular plug was performed, resulting in the disappearance of the shunt tract post-embolization. **(C,D)** A 10-year-old girl with preoperative angiography demonstrating a shunt between the left portal branch and the left hepatic vein. The shunt tract resolved after treatment with a vascular plug.

**Figure 2:**
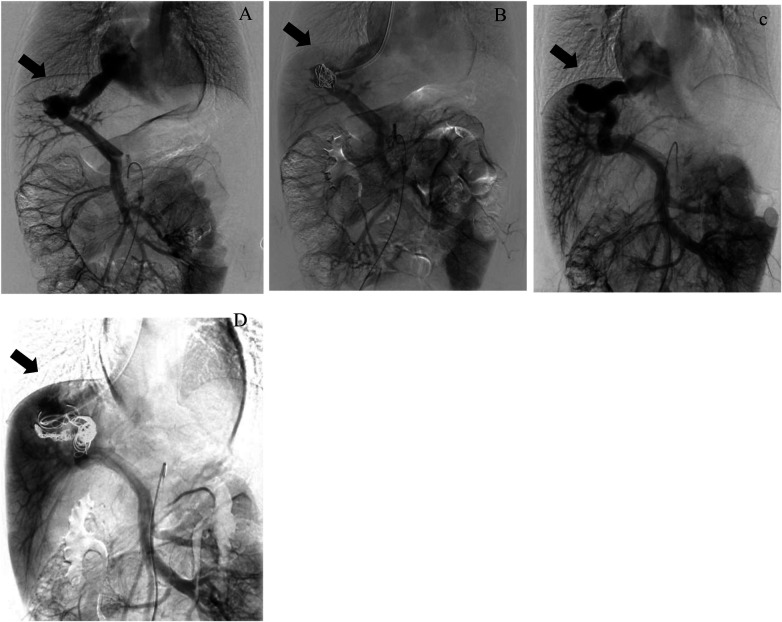
**(A,B)** A 3-year-old boy with intrahepatic portal-systemic shunt, where the right hepatic branch diverged into the right hepatic vein. The tortuous shunt tract made vascular occlusion difficult. After coil embolization, the shunt improved, and the desired effect was achieved as the thrombus advanced further. **(C,D)** A 6-year-old boy with intrahepatic portal-systemic shunt, where the right hepatic branch diverged into the right hepatic vein. The shunt tract resolved after coil embolization.

**Figure 3:**
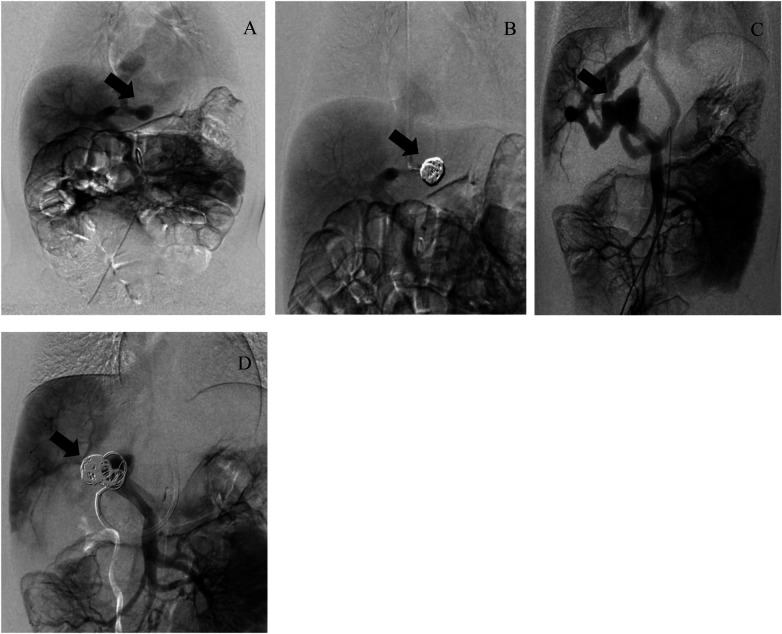
**(A,B)** A 1-year-old girl with intrahepatic portal-systemic shunting accompanied by venous cystic dilation, which resolved after coil embolization. **(C,D)** A 3-year-old boy with multiple intrahepatic shunts accompanied by venous cystic dilation, which resolved after coil embolization.

**Figure 4:**
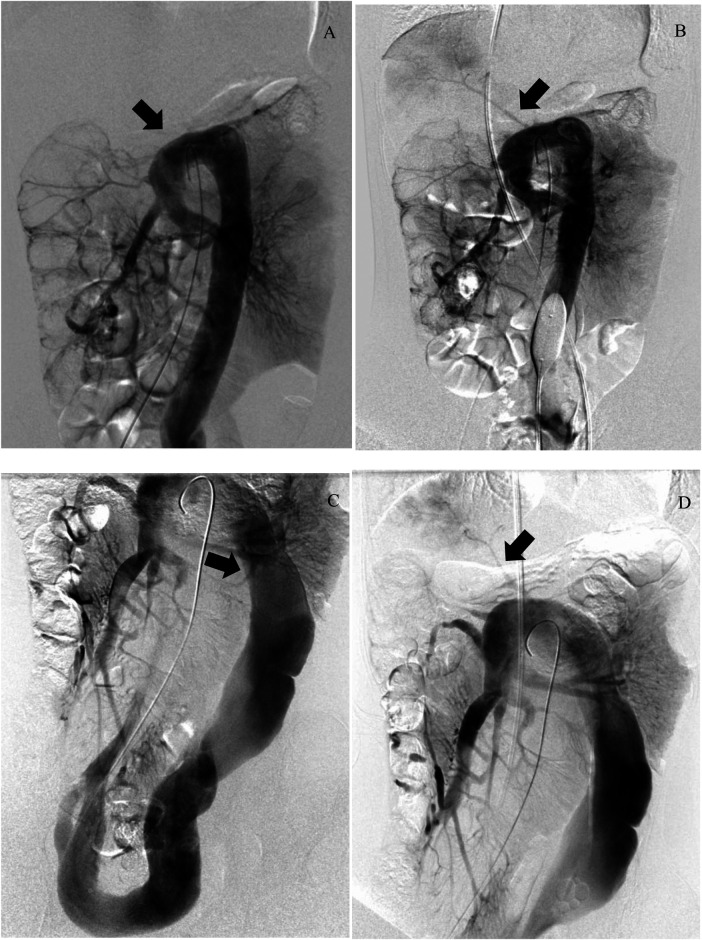
**(A,B)** A 5-year-old girl with extrahepatic portal-portal shunt accompanied by lower gastrointestinal bleeding. Intraoperative angiography revealed tortuous and thickened shunt channels, and after balloon occlusion of the shunt, fine portal veins were visualized. **(C,D)** A 13-year-old girl with extrahepatic portal-portal shunt accompanied by lower gastrointestinal bleeding. Similarly, only fine portal veins were visible, and the intrahepatic portal vein was not visualized.

**Figure 5:**
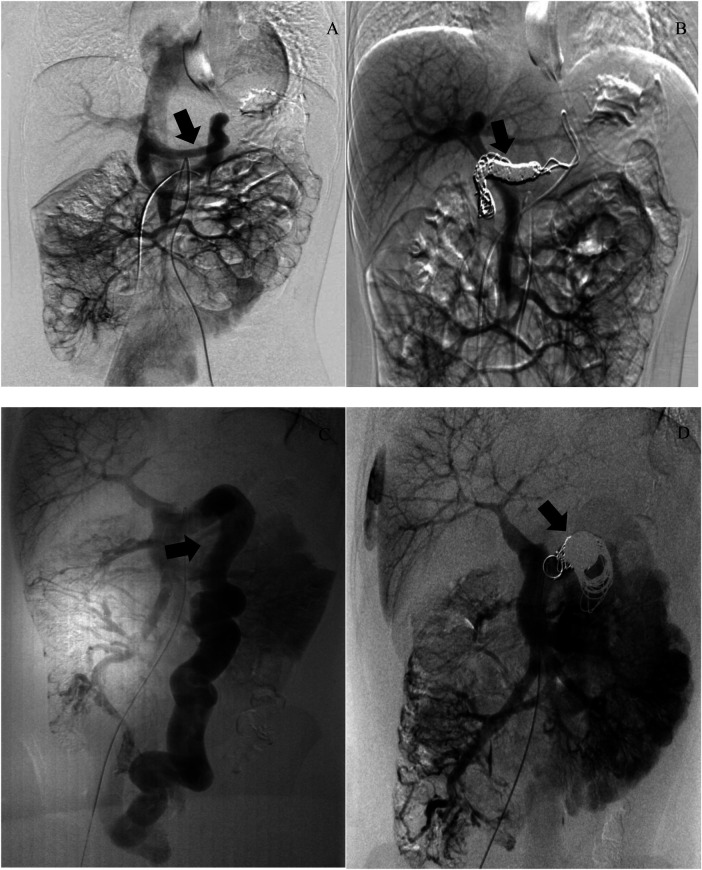
**(A,B)** A 5-year-old boy with extrahepatic portal-portal shunt and superior mesenteric vein-to-left renal vein shunt, which resolved after coil embolization. **(C,D)** A 5-year-old boy with extrahepatic shunt and superior mesenteric vein-to-right iliac vein shunt, with well-developed intrahepatic portal system, which resolved after coil embolization.

1. Intrahepatic portal-systemic shunt (17 cases): All pediatric patients underwent femoral artery puncture first. Upon successful intervention, the superior mesenteric artery was selectively accessed via ultrasound to perform indirect portal venography. The shunt tract and intrahepatic portal development were assessed based on venographic findings. Subsequently, right internal jugular vein puncture was performed, and the shunt tract was selectively entered via ultrasound to complete a temporary balloon occlusion test. Portal pressure measurements were conducted to determine the feasibility of complete occlusion. Different embolic materials were selected according to the type of shunt tract: Amplatzer occluders were prioritized for straight, single-branch shunts (Figure 1), while coil embolization was preferred for tortuous shunts (Figure 2). For cases with tumor-like dilation, coil embolization alone was employed. The decision to combine Amplatzer occluder therapy was based on indirect portal venography results (Figure 3).
2. Abernethy malformation type I (6 cases): All pediatric patients underwent femoral artery puncture first. After successful intervention, the superior mesenteric artery was selectively accessed via ultrasound to perform indirect portal angiography. Based on the angiographic findings, the shunt tract and intrahepatic portal development were assessed. Subsequently, right internal jugular vein puncture was performed, and the shunt tract was selectively entered via ultrasound to complete the temporary balloon occlusion test. In these 6 cases, intrahepatic portal hypoplasia was identified during indirect portal angiography. During the temporary balloon occlusion test, portal pressure was significantly elevated (exceeding 26 cmH_2_O) after occlusion of the shunt tract. Additionally, indirect portal angiography performed under balloon occlusion confirmed intrahepatic portal hypoplasia (Figure 4). Consequently, interventional embolization was not performed.
3. Abernethy malformation type II (2 cases): All pediatric patients underwent femoral artery puncture first. After successful intervention, the superior mesenteric artery was selectively accessed via ultrasound to perform indirect portal angiography. The shunt tract and intrahepatic portal development were assessed during the portal phase angiography. Subsequently, right internal jugular/femoral vein puncture was performed to selectively enter the shunt tract, completing the temporary balloon occlusion test. In these two cases, well-developed intrahepatic portal vessels were already visualized during indirect portal angiography, with portal pressure ≤26 cmH_2_O during the balloon occlusion test, meeting the criteria for embolization. Due to tortuous shunt tracts, both patients underwent coil embolization (Figure 5).

#### 1.2.4 Efficacy and follow-up

1. Technical success criteria: shunt occlusion rate ≥90%, with post-closure PVP ≤26 cmH_2_O, and no severe postoperative complications.
2. Follow-up: Postoperative follow-up at 1, 3, 6, and 12 months, including liver function tests, blood ammonia levels, and portal vein contrast-enhanced CTA or ultrasonography to evaluate the shunt tract status.

#### 1.2.5 Statistical methods

Statistical analysis was performed using SPSS 26.0. Measurement data were expressed as (mean ± standard deviation), and count data as percentages (%). Preoperative and postoperative liver function, coagulation, and blood ammonia levels were compared using paired *t*-tests, with *P* < 0.05 considered statistically significant.

## 2 Results

### 2.1 Success rate and complications of interventional therapy techniques

The success rate of interventional therapy in 26 pediatric cases was 73% (19/26). Among them, the technical success rate in the intrahepatic portal-systemic shunt group was 94.4% (17/18), while the Abernethy malformation technique achieved a success rate of 25% (2/8). Specifically, 6 cases in the Abernethy malformation group failed, and 1 case in the intrahepatic portal-systemic shunt group failed. The reasons were intraoperative angiography revealed poor development of the intrahepatic portal vein, and post-blocking measurement showed a significant increase in portal pressure, rendering interventional surgery unnecessary.

One case of mild postoperative complication occurred, presenting as a local hematoma due to bleeding at the percutaneous liver puncture site. The condition did not worsen after active conservative treatment. No severe complications were observed in all pediatric patients.

### 2.2 Improvement in liver function and blood ammonia levels

There were no statistically significant differences in ALT and AST levels between preoperative and postoperative periods (*P* > 0.05) (Table 2). Bile acid levels showed significant improvement postoperatively (*P* < 0.01) (Table 3). Serum ammonia levels decreased from 48.52 ± 18.76 μmol/L preoperatively to 30.63 ± 10.58 μmol/L postoperatively (*P* < 0.01) (Table 4).

**Table 2:** Liver function indicators before and after surgery (U/L).

Liver function indicators	Preoperative (*x* ± *s*)	Postoperative (*x* ± *s*)	*P*-value
ALT (U/L)	25.62 ± 10.25	23.15 ± 10.83	0.13
AST (U/L)	40.58 ± 15.67	39.82 ± 14.95	0.66

**Table 3:** Bile acid levels before and after surgery (μmol/L).

Indicators	Preoperative (*x* ± *s*)	Postoperative (*x* ± *s*)	*P*-value
Total bile acids	68.42 ± 51.23	25.78 ± 39.45	0.0003

**Table 4:** Pre-and postoperative blood ammonia levels (μmol/L).

Indicators	Preoperative (*x* ± *s*)	Postoperative (*x* ± *s*)	*P*-value
Blood ammonia	48.52 ± 18.76	30.63 ± 10.58	0.0001

### 2.3 Diversion channel condition

Postoperative follow-up CTA/ultrasound revealed a recanalization rate of 5.2% (1/19), attributed to abnormal dilation of the shunt tract, increased blood flow, and incomplete occlusion by the coil and thrombus, leading to recanalization.

## 3 Discussion

In this study, the overall success rate of CPSS interventional therapy was 73%, with intrahepatic portal shunt success rate at 94.4% and extrahepatic portal shunt success rate at 25%. The significant difference in success rates between these two types of portal shunts is attributed to the development of intrahepatic portal vessels. The study found that the age of children who could not undergo interventional embolization was not concentrated, indicating that age and disease duration cannot serve as indicators for assessing disease severity. Regarding complications, the incidence rate of 3.8% confirmed the safety of interventional therapy in pediatric CPSS. For intrahepatic portal shunts, anatomical characteristics eliminate the need for percutaneous liver puncture to access the portal vein, thereby avoiding complications such as hemorrhage. In terms of short-term efficacy, the recovery rate of liver function was 63%, the improvement rate of bile acids was 73.7%, and the improvement rate of blood ammonia was 100%, suggesting that interventional therapy can rapidly improve hepatic perfusion and metabolism.

In this study, all pediatric patients achieved normal blood ammonia levels postoperatively. Although blood ammonia levels are proportional to shunt extent and normalize after shunt tract occlusion, studies have demonstrated that this correlation does not always correlate with the severity of hepatic encephalopathy (2, 3). Kudo et al. (4) demonstrated that shunt rates <24%–30% do not induce hepatic encephalopathy, even in cirrhotic patients, whereas shunt rates exceeding 60% increase the risk of encephalopathy and hepatic dysfunction in patients of any age, necessitating active intervention. In this study, no statistically significant differences were observed in pre-or postoperative liver function parameters among the pediatric patients. Given that most congenital portal-systemic shunts were detected early and did not cause severe hepatic impairment, early identification and prompt treatment of portal-systemic shunts play a critical role in improving patient prognosis. Additionally, long—term follow—up is equally essential. It is necessary to comprehensively evaluate the disease progression of these children 5 years, 10 years, and even longer after the operation. Furthermore, for the children who underwent interventional embolization, whether the embolic materials placed in the body will have an impact on the children at different developmental stages also demands further follow—up and assessment.

The selection of embolization materials should be determined based on the type of shunt and intraoperative conditions. For straight shunt tracts without multiple branches, Amplatzer occluder embolization is preferred. It is recommended to perform indirect portal venography again before releasing the occluder to further confirm the embolization status and avoid residual shunts due to inappropriate occluder placement. For multiple shunts with tortuous tracts or significant tumor-like dilation, coil embolization is preferred to achieve optimal occlusion of the shunt tract and prevent residual shunts. Combining coil embolization with Amplatzer occluder can further enhance the occlusion effect.

In this study, all pediatric patients received routine low molecular weight heparin (LMWH) anticoagulation postoperatively. Follow-up portal vein ultrasound revealed no evidence of portal thrombosis or portal hypertension. Therefore, active anticoagulation therapy should be administered to all pediatric patients with any embolic material postoperatively, with close monitoring of coagulation function to prevent complications such as portal vein thrombosis, mesenteric vein thrombosis, thrombus detachment, or portal hypertension. Postoperative portal vein ultrasound should be actively performed to detect potential portal hypertension early. Liao (5) et al. observed in 592 subjects that LMWH and warfarin could reduce the incidence of portal vein thrombosis (PVST) in patients with splenectomy-associated liver cirrhosis without increasing bleeding risk, further clarifying the importance and safety of postoperative anticoagulation in portal vein surgery.

In this study, interventional therapy was unsuccessful in all children with Abernethy malformation type I, with some subsequently undergoing surgical intervention. Some researchers have proposed the preliminary placement of stents within the shunt tract, which, due to thrombus formation within the stent, can reduce the shunt diameter and achieve effects equivalent to partial surgical ligation. However, the safety and efficacy of this approach still require large-scale data validation. For children with Abernethy malformation type II, surgical treatment has been the conventional approach. Zhang et al. (6) reported that among 12 cases of Abernethy malformation type II treated surgically, hematochezia symptoms were alleviated in 9 patients postoperatively. Bilirubin and transaminase levels returned to normal in 2 patients, while oxygen saturation normalized in 1 patient. All patients exhibited normal postoperative blood ammonia levels. However, surgical procedures require open abdominal incisions, increasing patient discomfort and hospitalization time, and have gradually been replaced by interventional therapy.

Research on novel embolic materials is crucial for pediatric treatment. Currently, both Amplatzer occluders and coils are metallic implants requiring long-term retention in the body. Wang and colleagues (7) conducted a multicenter randomized controlled trial using a novel fully bioresorbable occluder to seal pericardial ventricular septal defects via catheterization. The study demonstrated that the bioresorbable occluder can be safely implanted with safety and efficacy comparable to metallic occluders. However, its limitation lies in the potential for incomplete degradation, which may lead to new complications.

Limitations of this study: single-center design, small sample size, lack of control group, short follow-up duration, and insufficient medium-to-long-term liver fibrosis data. Future research should expand the sample size, implement multicenter collaboration, extend follow-up duration, and refine the efficacy evaluation system.

## 4 Conclusion

Anatomical subtype-based individualized intervention strategies for pediatric CPSS are safe and effective, with definitive short-term efficacy. Postoperative follow-up demonstrated significant improvements in liver function and blood ammonia levels, with no severe complications occurring.

## Data Availability

The datasets presented in this study can be found in online repositories. The names of the repository/repositories and accession number(s) can be found in the article/Supplementary Material.
